# Sex Differences in Participation and Performance Trends in Time-Limited Ultramarathon Events

**DOI:** 10.1155/tsm2/1129276

**Published:** 2024-12-23

**Authors:** Mabliny Thuany, Thayse Natacha Gomes, Elias Villiger, Pantelis T. Nikolaidis, Volker Scheer, Katja Weiss, Thomas Rosemann, Beat Knechtle

**Affiliations:** ^1^Department of Physical Education, State University of Para, Belém, Pará, Brazil; ^2^Department of Physical Education, Federal University of Sergipe, São Cristóvão, Sergipe, Brazil; ^3^Institute of Primary Care, University of Zurich, Zurich, Switzerland; ^4^School of Health and Caring Sciences, University of West Attica, Athens, Greece; ^5^Ultra Sports Science Foundation, Pierre-Benite, France; ^6^Medbase St. Gallen Am Vadianplatz, St. Gallen, Switzerland

**Keywords:** age-related differences, endurance, exercise, performance, running, sex gap

## Abstract

**Aim:** We aimed to analyze sex differences in time-limited ultramarathon participation, while also identifying trends in participation, age, and performance across different formats of events, from 1990 to 2020.

**Method:** This is an exploratory study, using data obtained from the official event web pages. We downloaded information regarding the year of the event, athletes' year of birth, sex, race event, ranking, and mean running speed (km/h). The sex gap in participation was presented through equiplots. Regression models were fitted to analyze trends in participation, age, and performance, considering a 95% confidence interval.

**Results:** A similar pattern of increase in participation and age was shown for athletes of both sexes until 2019. The sex gap remained, displaying different patterns across race events. A general trend of slower mean running speeds was shown. The random-effects analysis showed that sex and age played significant roles in the performance trajectory across the years, in each race event.

**Conclusions:** Apart from the 8-day race among females, there was a decline in the performance across all race durations. Despite the trend of performance decline, future studies need to address the relevance of this decline in both theoretical and practical terms.

## 1. Introduction

The rise in mass sports, especially in running events, has been highlighted over the last years [1]. Data covering 107.9 million results from more than 70 thousand events worldwide revealed a peak in the number of runners in 2016, with an increase in participation of 57% between 2008 and 2018 [1–4]. Although this rise is observed across race distances of 5 km, 10 km, half-marathon, and marathon, the growth in ultramarathon participation has been highlighted [5]. Ultramarathons are races with a distance higher than a marathon, being categorized as “distance-limited” or “time-limited,” performed in one or multiple days [6]. Global trends suggest a tripling number of ultramarathons over the last 30 years [7], which can be linked to factors such as the desire for social interaction and health benefits [8].

Despite previous studies reporting the increase in the number of female participants in ultramarathon events [9], it is well known that women are underrepresented in mass sports events, increasing the researchers' interest in investigating sex differences [10–12]. Sex differences have been remarkable in physical activity and sports practice [13, 14], being connected to socioeconomic and environmental factors [15] and embedded in countries' values, norms, and culture. Despite previous studies addressing participation rates among runners of both sexes [16], understanding the participation gap using the lenses of inequalities advances the theoretical discussion and also helps to provide insights for future studies and political initiatives.

Beyond the analysis of participation, performance trends have been studied. However, the first important challenge is to define performance and to provide a practical meaning for the concept. Despite the lack of clarity regarding the theoretical concept, a previous study pointed out that in long-distance running, performance has been considered as an outcome, measured through the running event indicators, such as the finish time, ranking position, or average speed [17]. Among the different indicators, the average speed has been one of the most used [18]. By focusing on the average speed, researchers can obtain a clear and concise representation of a runner or group performance, facilitating comparison between studies, age groups, sex, competitive levels, and also race distances. Also, it is important information for coaches, since it can be manageable during training periodization, despite the lack of control for the influence of different terrain and altitudes on the runners' speed.

For example, in the realm of ultra-endurance running, it has also been noted that in 6-h events, athletes slowed down about 0.18 km/h over the years [17], which is similar to the trend related to the reduction in speed of 1.37 km/h and 4.65 km/h among men competing from 1971 to 2020 in 12-h and 24-h events, respectively. Despite this information, most of the studies are developed considering specific distances or time, such as 6 h, 12 h, 24 h, and 161 km, with a lack of information about participation gap and performance trends considering different distance- and time-limited events [19]. In this sense, covering a big portion of ultra-endurance events, our purpose was to analyze sex differences in ultramarathon participation, while also identifying trends in participation, age, and performance across different formats of time-limited events (6-h, 12-h, 24-h, 48-h, 72-h, 6-day, 8-day, and 10-day). Based on the existing literature [20], we hypothesized an increase in participation and age and a decrease in performance irrespective of the race duration.

## 2. Methods

### 2.1. Ethical Approval

The Institutional Review Board of St Gallen, Switzerland, approved this study (EKSG 01/06/2010). All methods comply with the recommendations for the seventh revision of the Declaration of Helsinki. Since the study involved the analysis of publicly available data, the requirement for informed consent was waived.

### 2.2. Design and Sample

This is an exploratory study using information obtained from the event's official web pages of Deutsche Ultramarathon Vereinigung (DUV) (https://statistik.d-u-v.org/geteventlist.php). Data were collected from available official results for athletes of both sexes and participants in time-limited events (6-h, 12-h, 24-h, 48-h, 72-h, 6-day, 8-day, and 10-day) between 1807 and 2020. Downloaded information included the year of the event and race event, and participants' information such as date of birth, sex, athlete ranking, average running speed (km/h), and country of residence. The athlete's age was computed, considering the year of birth and the year of the competition. Considering the lower participation rate until 1989 (10,477 runners), during data cleaning and analysis, we excluded those competing before 1990 (male: 88%; female: 12%; 6-h: 4.3%; 12-h: 8.4%; 24-h: 70.5%; 48-h: 8.4%; 72-h: 0.2%; and 6-day: 8.2%).

### 2.3. Variables Analyzed

Performance was quantified in terms of race average speed (in km/h). The decision to use speed as the outcome variable was based on methodological limitations (lack of information about personal best time, for example), practical applications, and previous literature [18].

### 2.4. Independent Variables

Sex was dichotomized into male and female (based on the available information from the races' webpage), while age and years were considered continuously.

### 2.5. Statistical Analysis

Descriptive statistics were computed using percentages, means, and standard deviations. Data normality was tested using the Kolmogorov–Smirnov test by sex and race event. A linear regression analysis was estimated to verify trends of participation and age, over years, in both sexes. The sex gap in participation was represented using equiplots. Equiplots are used to represent health inequalities [21], in which dots represent the participation prevalence by sex, whereas the lines indicate the gap between them. Graphics were stratified by race event and created through the International Center for Equity in Health (https://equidade.org/equiplot_creator).

Following, we fitted a mixed-effects regression to estimate the association between sex, age, year, and performance (i.e., speed) in each race event. In addition, random effects were estimated, accounting for potential variability in the effects of the year across different sexes and ages. For more detailed information about performance trends over the years, we used the Cochran–Armitage test and Mantel test to verify if the trend is significant. Average speed values were graphically presented. Statistical analysis was performed in Stata (Version 14), GraphPad Prism 8, and WinPepi, adopting a confidence interval of 95%.

## 3. Results

The total sample comprised 373,186 athletes (75.3% of males, *n* = 281,104%; 24.7% of females, *n* = 92,082), from 134 countries. The highest frequency of participation was shown for 24-h events (*n* = 145,671; 39%), followed by 6-h events (*n* = 109,955; 29.5%), while the lowest frequency of participation was shown for those competing in 10-day (*n* = 838, 0.2%) and 8-day (*n* = 852, 0.2%) events.  Table 1 presents athletes' descriptive information, including participation by race event, average age, and running speed, by sex. Among females, most of the athletes participated in 24-h (39.6%) and 12-h (28.5%) events, while among men, 24-h (38.9%) and 6-h (30.9%) events were the most popular. For both sexes, the lowest participation was observed in 8-day (0.2% for both sexes) and 10-day (0.3% and 0.2% for female and male, respectively) race events. Also, for both sexes, the highest age mean values were shown for athletes competing in 8-day events, while the fastest running speeds were shown for athletes competing in 6-h events.

**Figure 1:**
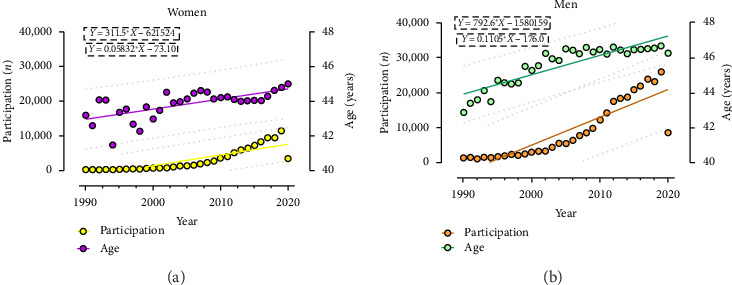
Participation and average age trends over time, for both sexes.

A linear trend of participation and age was shown (Figure 1). Participation rates ranged from 1170 (1992) to 25,918 (2019) among men and from 209 (1992) to 11,465 (2019) among women, with a similar pattern of increased participation. The slope results of the linear regression showed that, over time, men presented a higher increase in participation (*Y* = 792.6 ∗ *X*–1,580,159), compared to women (*Y* = 311.5 ∗ *X*–621,524). In both sexes, participation declined in 2020. Regarding age, mean values ranged from 42 to 46 years in men, while in women it ranged from 41 to 45 years. The regression analysis showed a trend of increased age over the years.

**Figure 2:**
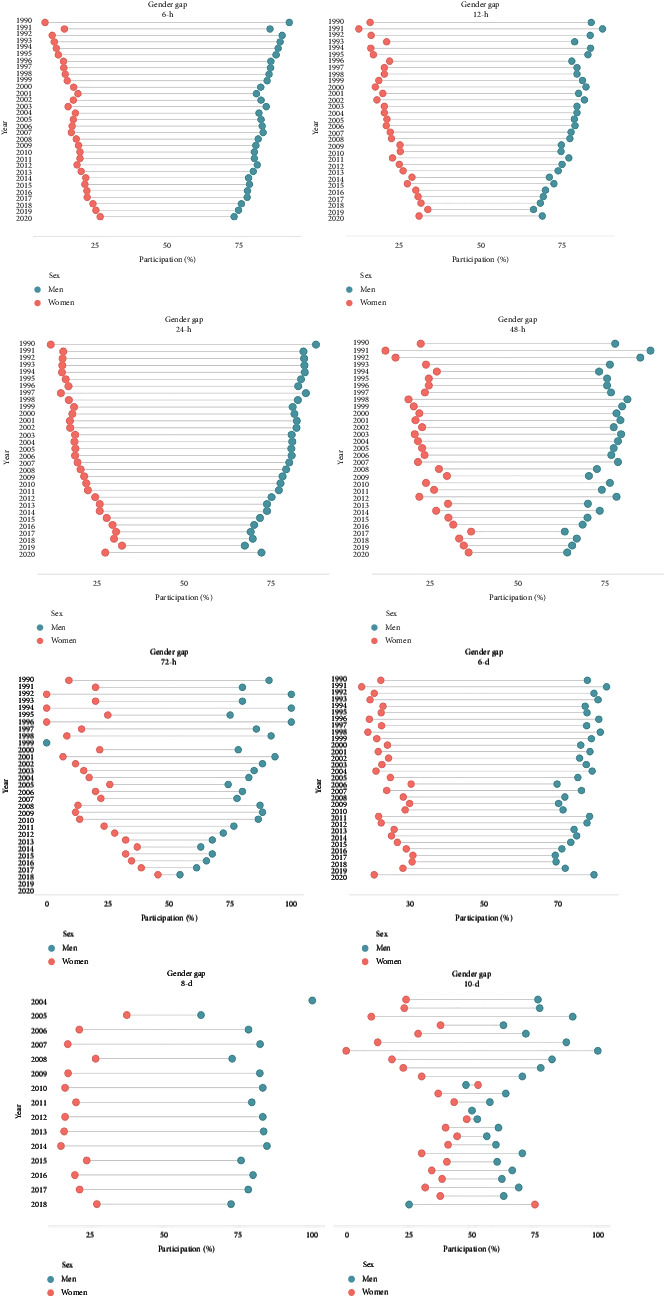
Sex gap for participation in ultra-endurance events.

Figure 2 shows the sex gap in participation. The pattern of the funnel plot is the predominant pattern within the different distances, particularly for 6-h and 8-h events. These patterns highlight a reduction in the gap over time, with more females finishing the events over the last years, compared to the first years. For participants in 72-h events, a different pattern emerges, indicating an erratic trend of participation between both sexes, over time. For 6-day events, the gender gap trends remained stable, which visually differs from those competing in 8-day and 10-day events.

Tables 2 and 3 provide coefficients and 95% confidence intervals for sex, age, and year across different events. For the fixed part of the model, except for runners competing in 72-h events, sex and age were significant predictors for the remained distances, with males performing better than females in these races. For age, a significant association was shown for ultramarathoners competing in 6-h (*β* = −0.02; 95% CI = −0.02 to −0.02), 12-h (*β* = −0.01; 95% CI = −0.01 to −0.01), 24-h (*β* = −0.006; 95% CI = 0.008 to 0.005), 48-h (*β* = −0.005; 95% CI = −0.008 to −0.003), and 10-day (*β* = −0.017; 95% CI = −0.02 to −0.01) events, with older runners performing worse. Over the years, a trend of slower mean running speed was shown for most of the race events. A trend of increased performance was observed for ultramarathoners competing in 8-day events. No significant association was found for those in 10-day events.

**Figure 3:**
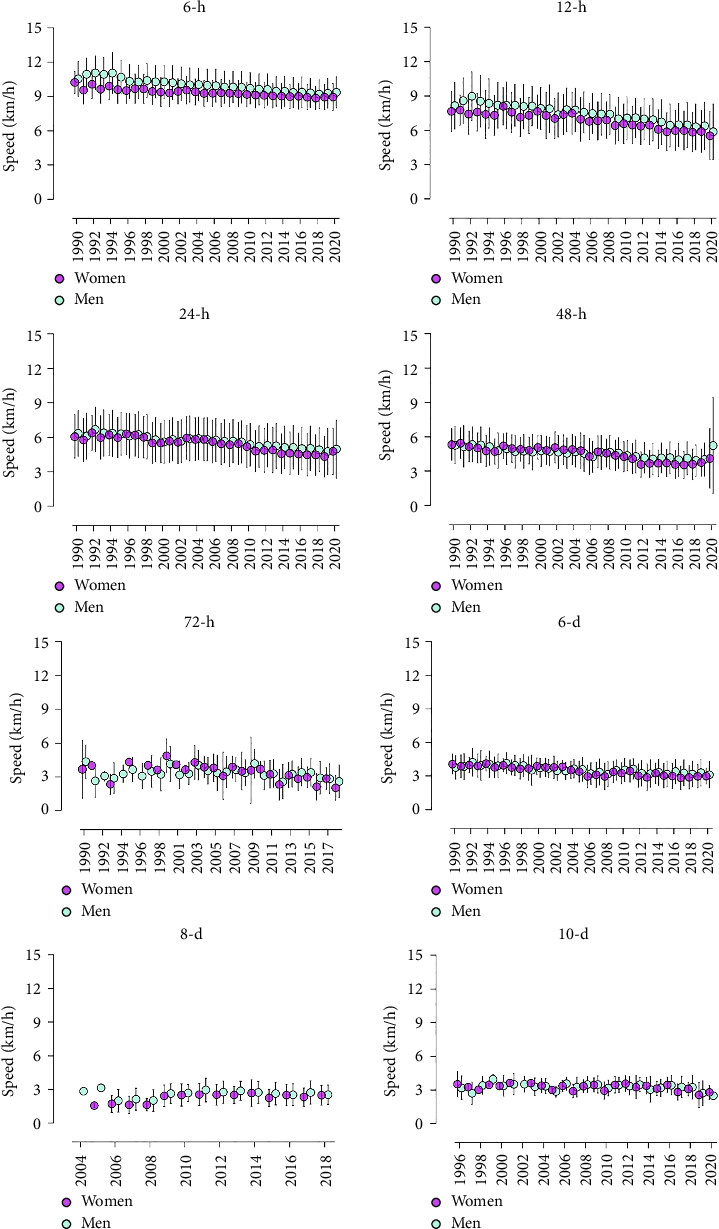
Average speed values over the years, categorized by sex and race events. Differences in the *x*-axis range and data are due to the missing data or different time ranges: 6-h (female and male: range from 1990 to 2020); 12-h (female and male: range from 1990 to 2020); 24-h (female and male: range from 1990 to 2020); 48-h (female and male: range from 1990 to 2020); 72-h (female: range from 1990 to 2018, with missing data in 1992, 1994, 1996, and 1999 years; male: range from 1990 to 2018 with missing data in 1999); 6-day (female and male: range from 1990 to 2020); 8-day (female: ranging from 2005 to 2018, with missing data between 1990 and 2003 years; male: ranging from 2004 to 2018, with missing data between 1990 and 2003 years); 10-day (female: ranging from 1996 to 2020 with missing data between 1990, 1995, and 2002 years; men: ranging from 1996 to 2020 with missing data between 1990 and 1995 years).

For random effects, it was observed that sex and age play significant roles in the performance trajectory across the years, in each race event. The likelihood-ratio test showed that this model offered significant improvement over a linear regression model with only fixed effects, meaning that the intercepts were significantly different between sexes and different ages, in each race event. However, the variance of the residuals indicates a higher unexplained variability in performance after considering sex, age, and year.  Figure 3 presents speed mean values over the years. Apart from the 8-day race among females, all significant trends showed a decline in performance.

## 4. Discussion

We aimed to analyze sex differences in participation, age, and performance of ultramarathoners. Our main findings showed that the sex gap in participation remains among runners; athletes are getting older and slower over time, except for those competing in 8-day events. These results confirm our previous hypothesis.

### 4.1. Ultra-Endurance Sex Differences

A significant and positive trend of participation was shown for both sexes although the sex gap remains. These results support previous findings [20, 22], despite a decline in participation in 2020, which could be due to the COVID-19 pandemic. A more accurate analysis regarding the impact of COVID-19 on ultra-endurance events can be checked in a previous study developed by Scheer et al. [23]. The general trend of increasing the number of runners was previously related to a plethora of factors, such as the motivation to run as psychotherapy, life meaning, and the sense of belonging [24]. Running motivation includes health, quality of life, and desire to experience new challenges and has been considered an important factor for running practice. However, most of the studies about motives/motivation to run were developed by sampling runners used to covering distances shorter than the marathon, which supports the relevance of performing studies in the ultra-endurance context.

Regarding the higher proportion of males, these results confirm previous findings [25]. This sex gap has been observed among adolescents/young runners [24, 26], as can be observed in the study conducted by Scheer et al. [19], where authors reported higher participation of males among young runners in time-limited events, which suggests that the gap starts early. Beyond the context of sports practice, the sex gap in physical activity is a public health concern that should be considered strategically to change it. Previous studies highlight a high sex gap, ranging according to age, income, and country [15], which also impacts health outcomes. For instance, in Brazil—a country with continental dimensions and high economic disparity, a higher percentage of males (22.8%), compared to females (6.9%), report engaging in running as a leisure activity, and this difference varies according to age [27].

Nevertheless, it is important to emphasize the pattern of gap differences for those competing in 72-h and 10-day races, which differ from most other races. The explanation for these different patterns remains unclear. Notably, for both sexes, the 72-h race presents a lower number of participants, suggesting a reduced level of popularity compared to other race distances. This factor may contribute to the observed smaller participation gap. Considering that society and culture influence human behavior, with an emphasis on sex roles, future studies could investigate how the place where the races occur influences participation. Additionally, researchers could explore the perceived barriers and support influencing the participation of both sexes in these races, with special attention to the women's feelings and experiences. Considering mass sports events as a tool to increase physical activity levels and provide health benefits, strategies to promote participation among women should be studied and investigated in future studies, considering the profile and the needs of the participants, as well as the most popular race, and the place where the races are held.

### 4.2. Ultra-Endurance Trends: Older and Slower

Our main findings showed a decline in performance among most of the race events. These results follow previous studies. The decline in performance was previously presented for athletes competing in short-distance (e.g., 5 km and 10 km) and long-distance events (i.e., half-marathon and ultramarathon) [1, 3, 28]. Marathon statistics showed that between 2008 and 2018, the runners' pace became slower by approximately 3:55 min [1], while for ultrarunning, runners have slowed by 15% since 1996 [28]. Similarly, data from global statistics reported a performance decrease in several distances and time-limited events, which highlights the need to move beyond trends analysis and use different research strategies to understand factors influencing these trends.

Despite the trend of declining performance, an important aspect of these results pertains to the relevance of this decline, especially considering the small values of the beta coefficients. Evaluating the relevance of this decline requires an analysis of the potential explanation factors and also the influence on the future of ultramarathon events. The first important explanation factor is the decline in performance might be influenced by the increase in participation. More people have been engaged in time-limited races and, for instance, these events have become more massive compared to the past, where the participation was limited to runners who were “selected” due to their performance. Also, motivation to engage in ultra-endurance events can be related to social benefits, instead of performance outcomes [26, 29, 30]. These characteristics suggest a need to broaden the scope to address several research topics, including the health implications of participating in ultramarathon events, the costs associated with training and competition, and not solely focusing on performance trends.

A positive trend was shown for age, which is following previous findings. This rise in age can be related to the general pattern of performance decline [31, 32]. A report using 27,088 results for lifelong athletes showed a linear decline trend for different track and field disciplines, with a higher decline beyond the age of 70 for runners and sprinters [32]. A longitudinal approach that reports performance over time for sub-3-h marathoners during five consecutive calendar decades suggested the possibility of reducing the performance decline (< 7% decade) until 60 years of age with a training regime maintenance [33]. This suggestion can be verified with ultra-marathon runners, considering they are older than marathoners.

Despite previous discussions regarding the potential for women to outperform men in ultra-endurance running [34], our results do not support this hypothesis. In general, males tend to perform better, even though the performance gap narrows at longer distances. While factors such as physiological differences, pacing strategies, and race conditions may contribute to this trend [35], more research is needed to understand the complex interplay of these variables in ultra-endurance events. For example, despite sex differences, our results showed that female ultramarathoners competing in 8-day events improved performance over time. These results should be interpreted considering the magnitude of performance improvement and also the number of athletes competing in these events. Regarding the magnitude of performance, the graphical results for the average speed values show that in 2018, the finishers covered about 0.9 km more than those competing in 2005, which seems to be a small improvement over these 12 years. However, even the small improvement results should be interpreted in light of limitations such as the sample size. Among the studied events, the 8-day events are one of those with lower participation rates, which can be influenced by the limited number of competitions held at the distance. Another point is that athletes competing in 8-day events presented the lowest running speed (female: 2.2 ± 0.9 km/h; male: 2.5 ± 0.9 km/h) compared to other race events, which means that the range for improvement can be higher than for athletes competing in other events. In this sense, generalizations must be carefully considered given that the performance trends in 8-day race events were only investigated for participation and performance analysis in young runners [19].

### 4.3. Limitations, Strengths, and Suggestions

The present study has several limitations. As we used data publicly available, missing data, and accuracies of performance (i.e., runners' speed), distance, technical characteristics of these events (e.g., altitude, terrain, and elevation), and number of participants by year, especially in the events, are difficult to manage. Another limitation includes the lack of additional information regarding runners' profiles. For example, information regarding socioeconomic status could be helpful to researchers in understanding the profile of the runners, especially considering sex differences and age groups, providing more accurate information to stakeholders. We also did not control for multiple participation between years and race events. Another important aspect is the need to delve into the understanding of sex inequalities in endurance sports practice, especially adjusting for countries' economic characteristics, and opportunities to engage in sports practice from an early age.

Despite the limitations related to publicly available data, including the accuracy of the data, and the lack of information about finishers' backgrounds, important strategies to shape policies, identify underrepresented groups, and work strategically to adequate the competitions, championships, and products to attend to the needs of the public are important outcomes of this analysis. Based on the present findings and using different research designs, researchers can try to deeply understand the sex gap in ultramarathons, advancing the knowledge regarding participation, and sex gaps, and providing directions. Future studies should consider using mixed design to move beyond prevalence information and deeply understand this phenomenon and participants' perspectives about the facilitators and barriers to training and competing in ultramarathons.

## 5. Conclusion

Analyzing data from 1990 to 2020, it was shown that there was an increase in the number of participants of both sexes, although the sex gap remains. A positive trend was observed for age, which suggests that runners are getting older. Generally, runners are getting slower over the years, except those competing in 8-day events. Despite the trend of performance decline, future studies need to address the relevance of this decline in both theoretical and practical terms.

## Figures and Tables

**Table 1:** Total sample, age, and average speed values for ultramarathoners of both sexes, in each race event.

Race event	Female (*n* = 92,082)			Male (*n* = 281,104)		
	Total sample (%)	Age (mean (SD))	Speed (mean (SD))	Total sample (%)	Age (mean (SD))	Speed (mean (SD))
6-h	23,099 (25.1%)	43.2 (9.3)	9.0 (1.13)	86,856 (30.9%)	46.1 (10.1)	9.5 (1.3)
12-h	26,248 (28.5%)	43.4 (10.1)	6.1 (1.7)	68,296 (24.3%)	45.3 (10.9)	6.7 (2.0)
24-h	36,428 (39.6%)	44.9 (10.2)	4.8 (1.8)	109,243 (38.9%)	46.5 (11.0)	5.3 (1.9)
48-h	3597 (3.9%)	47.0 (10.7)	4.0 (1.4)	9286 (3.3%)	48.7 (11.5)	4.3 (1.6)
72-h	509 (0.6%)	46.9 (10.7)	2.7 (1.2)	1198 (0.4%)	49.0 (12.4)	3.1 (1.3)
6-day	1723 (1.9%)	47.5 (11.3)	3.2 (1.0)	5033 (1.8%)	49.5 (12.2)	3.4 (1.1)
8-day	175 (0.2%)	51.0 (10.9)	2.2 (0.9)	677 (0.2%)	51.9 (12.1)	2.5 (0.9)
10-day	303 (0.3%)	44.9 (11.8)	3.1 (0.8)	535 (0.2%)	46.3 (12.3)	3.2 (0.9)

*Note:* Age, years; speed, km/h.

Abbreviation: SD = standard deviation.

**Table 2:** Mixed-effects models to estimate the association between sex, age, year, and performance (i.e., speed in km/h) in time-limited events.

Outcome: speed in km/h	6-h	12-h	24-h	48-h	72-h
Independent variables	*β* (95% CI)	*β* (95% CI)	*β* (95% CI)	*β* (95% CI)	*β* (95% CI)
Sex	0.68⁣^∗^ (0.60; 0.76)	0.58⁣^∗^ (0.50; 0.66)	0.27⁣^∗^ (0.18; 0.35)	0.17⁣^∗^ (0.03; 0.32)	0.13 (−0.14; 0.41)
Age	−0.02⁣^∗^ (−0.02; −0.02)	−0.01⁣^∗^ (−0.01; −0.01)	−0.006⁣^∗^ (0.008; 0.005)	−0.005⁣^∗^ (−0.008; −0.003)	−0.0007 (−0.005; 0.004)
Year	−0.04⁣^∗^ (−0.05; −0.04)	−0.08⁣^∗^ (−0.08; −0.07)	−0.06⁣^∗^ (−0.06; −0.05)	−0.04⁣^∗^ (−0.05; −0.03)	−0.03⁣^∗^ (−0.05; −0.01)
Intercept	109.84 (99.86; 119.82)	175.65 (165.58; 185.71)	131.05 (120.50; 141.61)	96.19 (79.05; 113.33)	75.49 (39.85; 111.13)
Random effect					
Sex	0.01⁣^∗^ (0.01; 0.03)	0.01⁣^∗^ (0.00; 0.02)	0.24⁣^∗^ (0.01; 0.03)	0.05⁣^∗^ (0.03; 0.09)	0.12⁣^∗^ (0.06; 0.24)
Age	0.00⁣^∗^ (0.02; 0.04)	0.06⁣^∗^ (0.05; 0.07)	0.11⁣^∗^ (0.09; 0.12)	0.06⁣^∗^ (0.04; 0.09)	0.003⁣^∗^ (0.00; 0.00)
Residual	1.61 (1.59; 1.62)	3.43 (3.40; 3.46)	3.52 (3.49; 3.54)	2.29 (2.23; 2.35)	1.61 (1.50; 1.72)
Log-likelihood	−171845.21	−174599.58	−23542.899	−23542.899	−2799.0698
LR test vs. linear model: chi2 (2)	689.35	649.00	1661.18	291.31	54.65
Prob > chi2	< 0.001	< 0.001	< 0.001	< 0.001	< 0.001

*Note:* Sex (0 = female; 1 = male), age (continuous), and year (continuous).

⁣^∗^*p* < 0.05.

**Table 3:** Mixed-effects models to estimate the association between sex, age, year, and performance (i.e., speed in km/h) in 6-day, 8-day, and 10-day events.

Outcome: speed in km/h	6-day	8-day	10-day
Independent variables	*β* (95% CI)	*β* (95% CI)	*β* (95% CI)
Sex	0.19⁣^∗^ (0.11; 0.27)	0.31⁣^∗^ (0.12; 0.51)	0.085 (−0.06; 0.24)
Age	−0.009⁣^∗^ (−0.01; −0.006)	0.0002 (−0.006; 0.006)	−0.017⁣^∗^ (−0.02; −0.01)
Year	−0.03⁣^∗^ (−0.03; −0.02)	0.04⁣^∗^ (0.02; 0.07)	−0.005 (−0.01; 0.00)
Intercept	64.46 (54.750; 74.16)	−92.05 (−140.11; −44.00)	15.340 (−8.67; 39.35)
Random effect			
Sex	0.01⁣^∗^ (0.004; 0.02)	0.01⁣^∗^ (0.00; 0.09)	0.024⁣^∗^ (0.009; 0.06)
Age	0.04⁣^∗^ (0.02; 0.07)	0.13⁣^∗^ (0.06; 0.26)	07.11⁣^∗^ (0.00; 0.06)
Residual	1.07 (1.03; 1.11)	0.72 (0.62; 0.84)	0.67 (0.61; 0.74)
Log-likelihood	−9860.2122	−883.60053	−1033.9086
LR test vs. linear model: chi2 (2)=	53.14	13.89	10.50
Prob > chi2	< 0.001	0.001	0.005

*Note:* Sex (0 = female; 1 = male), age (continuous), and year (continuous).

⁣^∗^*p* < 0.05.

## Data Availability

The datasets generated during and/or analyzed during the current study are available from the corresponding author upon reasonable request.
